# Epithelial-mesenchymal transition in asthma: its role and underlying regulatory mechanisms

**DOI:** 10.3389/fimmu.2025.1519998

**Published:** 2025-01-22

**Authors:** Bingxi Zhang, Xinru Feng, Lincha Tian, Bo Xiao, Lixia Hou, Biwen Mo, Dong Yao

**Affiliations:** ^1^ Department of Pulmonary and Critical Care Medicine, The Second Affiliated Hospital of Guilin Medical University, Guilin, China; ^2^ The Laboratory of Respiratory Disease, Affiliated Hospital of Guilin Medical University, Guilin, China; ^3^ Guangxi Clinical Research Center for Diabetes and Metabolic Diseases, Guangxi Health Commission Key Laboratory of Glucose and Lipid Metabolism Disorders, The Second Affiliated Hospital of Guilin Medical University, Guilin, China; ^4^ Guangxi Key Laboratory of Metabolic Reprogramming and Intelligent Medical Engineering for Chronic Diseases, The Key Laboratory of Respiratory Diseases, Education Department of Guangxi Zhuang Autonomous Region, Guilin Medical University, Guilin, China

**Keywords:** asthma, airway inflammation, airway remodeling, epithelial-mesenchymal transition, mechanism, signaling pathways

## Abstract

Bronchial asthma (asthma) is a respiratory disease characterized by chronic inflammation, airway hyperresponsiveness, and airway remodeling. Numerous studies have delved into asthma’s pathogenesis, among which epithelial mesenchymal transition (EMT) is considered one of the important mechanisms in the pathogenesis of asthma. EMT refers to the transformation of epithelial cells, which lose their original features and acquire a migratory and invasive stromal phenotype. EMT contributes to normal physiological functions like growth, development, and wound healing. However, EMT is also involved in the occurrence and development of many diseases. Currently, the precise regulatory mechanism linking EMT and asthma remain obscure. Increasing evidence suggests that airway EMT contributes to asthma pathogenesis via dysregulation of associated control mechanisms. This review explores EMT’s significance in asthma and the regulatory networks associated with EMT in this context.

## 1 Introduction

Asthma is a respiratory disease characterized by chronic airway inflammation, leading to irreversible pathological remodeling of the airways as a result of repeated tissue damage and repair processes during long-term persistent inflammation. The prevalence of asthma is on the rise year by year, driven by urbanization and changing lifestyles (1). Currently, conventional asthma treatments possess certain limitations, as long-term steroid use carries significant side effects and only provides temporary symptomatic relief, rather than, a curative solution (2–4). Hence, there is an urgent need to delve deeper into the molecular mechanisms underlying asthma pathogenesis and develop novel therapeutic targets and treatment approaches.

Epithelial-to-mesenchymal transition (EMT) pertains to the biological process wherein epithelial cells undergo a phenotypic shift, losing their distinct epithelial traits and adopting a mesenchymal phenotype (5). This transition was first documented by Elizabeth Hay in the early 1980s, who noticed phenotypic transformations in the primitive stripes of chick embryos (6). Hay postulated that epithelial cells have the potential to downregulate their epithelial characteristics and acquire mesenchymal properties. This differentiation process, termed epithelial mesenchymal transition, contrasts with the reverse process, known as mesenchymal epithelial transition (MET). EMT is typically characterized by heightened cell migration, invasiveness, enhanced resistance to apoptosis, and modification in the extracellular matrix (ECM) composition (7).

Despite considerable research efforts and advancements achieved by many scholars in recent years, the precise mechanisms underlying EMT in asthma are still not fully elucidated. This review aims to consolidated the latest research to delve into the significance of airway EMT in asthma and the regulatory mechanisms involved, thereby enhancing our understanding of this complex process.

## 2 EMT in development and disease

EMT plays a pivotal role in growth and development, and it is also involved in tissue repair, organ fibrosis, and cancer progression from a pathological perspective. EMT is generally categorized into three types. Type I EMT is primarily relates to embryo implantation, embryonic development, and multiorgan formation (8). Type III EMT relates to metastasis of epithelial-derived cancers and the invasion of the surrounding stroma (9).

Type II EMT is associated with tissue repair responses, such as fibrosis. To heal damaged tissue, epithelial cells under-go EMT to transform into myofibroblasts, enabling tissue repair and healing. This process is also referred to reparative fibrosis. However, during persistent chronic inflammation, abnormal myofibroblasts formation can lead to progressive fibrosis. This result in organ parenchymal damage and multi organ fibrosis, including lung, liver, and kidney, due to excessive extracellular matrix (ECM) deposition (10). Consequently, EMT can contribute to tissue fibrosis, representing the pathological healing process involved chronic inflammation. Furthermore, the type II EMT discussed in this review is the primary subtype responsible for bronchial epithelial injury leading to airway remodeling in asthma (11).

## 3 The role of EMT in asthma

### 3.1 EMT and airway inflammation

Chronic exposure to harmful external factors prompts the airway epithelium to regulate inflammatory or immune responses through interaction with inflammatory cells and the release of cytokines and growth factors (e.g., epidermal growth factor (EGF) and transforming growth factor (TGF-β)), promoting airway EMT and subsequent structural alterations and remodeling of the airway wall (12, 13). Additionally, some studies have reported a correlation between eosinophils counts, the severity of airway remodeling in asthma and TGF-β1expression (14, 15). A study by Yasukaw et al. assessed the effect of eosinophils on EMT by infusing and co-culturing bronchial epithelial cells in mice infused with eosinophils (16). Results indicated that eosinophils exacerbated airway inflammation and fibrosis, exhibited EMT characteristics, and were associated with elevated TGF-β1 concentrations. These findings suggest that eosinophils induce fibrotic changes and EMT in airway epithelial cells through TGF-β1 secretion, thereby advancing airway remodeling in asthma. Doerner et al. investigated the role of inflammation in EMT, demonstrating a synergistic effect between TGF-β and IL1-β in an vitro model (3). Combined induction of TGF-β and IL-1β significantly altered e-cadherin expression and ECM component tenascin C. These results suggest that inflammatory milieu plays a critical role in EMT.

### 3.2 EMT and airway remodeling

Airway remodeling in asthma is driven by chronic airway inflammation, leading to irreversible pathological changes due to repeated damage and repair of airway tissue. This process involves several structural alterations, including epithelial destruction (17), thickening of reticular basement membrane associated with subepithelial fibrosis (18), and ECM deposition (19). When exposed to pathogens or toxic substances, epithelial cells can rebuild themselves upon damaged, but abnormal chronic inflammation and excessive harm can trigger tissue fibrosis. Fibrosis is hallmarked by excessive proliferation and activation of fibroblasts and myofibroblasts, leading to an overproduction and abnormal deposition of ECM (20). EMT emerges as a pivotal mechanism driving the proliferation and activation of fibroblasts and myofibroblasts (21). Iwano et al. demonstrated that epithelial cells can undergo EMT to form fibroblasts during pathological tissue fibrosis, representing a novel phenotypic reversal of cell fate (22). This phenotypic transformation underscores the potential plasticity of epithelial cell differentiation in mature tissues under pathological conditions. A study conducted by Johnson et al. further reinforced the significance of the EMT process in asthma-related subepithelial fibrosis, highlighting the plasticity of the airway epithelium in allergic airway disease (23).

### 3.3 EMT and immunity

In response to microorganisms, respiratory viruses, air pollutants, and allergens, the airway epithelium orchestrates the lung’s immune response by facilitating the activation, recruitment, and mobilization of immune cells. The typical inflammatory response of airway epithelial cells involves the release of cytokines such as IL-25, IL-33, and thymus stromal lymphopoietin (TSLP). These cytokines can trigger a downstream cascade involving the recruitment and activation of specific immune cells, while promoting the production of helper T cell 2 (Th2) cytokines (12, 24).

Beyond responding to injury, epithelial cells initiate repair processes that are crucial for driving airway remodeling. Activated epithelial cells and innate immune cells release pro-remodeling factors, including EGF and TGF-β, which stimulate EMT and fibroblast activation. This process promotes cell migration and contributes to the formation of a provisional extracellular matrix barrier (25). A Study has demonstrated that, in addition to fibroblasts, endothelial cells and smooth muscle cells, helper Th2 cell-mediated chronic inflammation leads to elevated levels of TGF-β1 in the airway, primarily through the aggregation of eosinophils and macrophages. TGF-β1, sequestered in the extracellular matrix, induces EMT and contributes to subepithelial fibrosis (23).

## 4 Regulation and control of EMT

**Figure 1:**
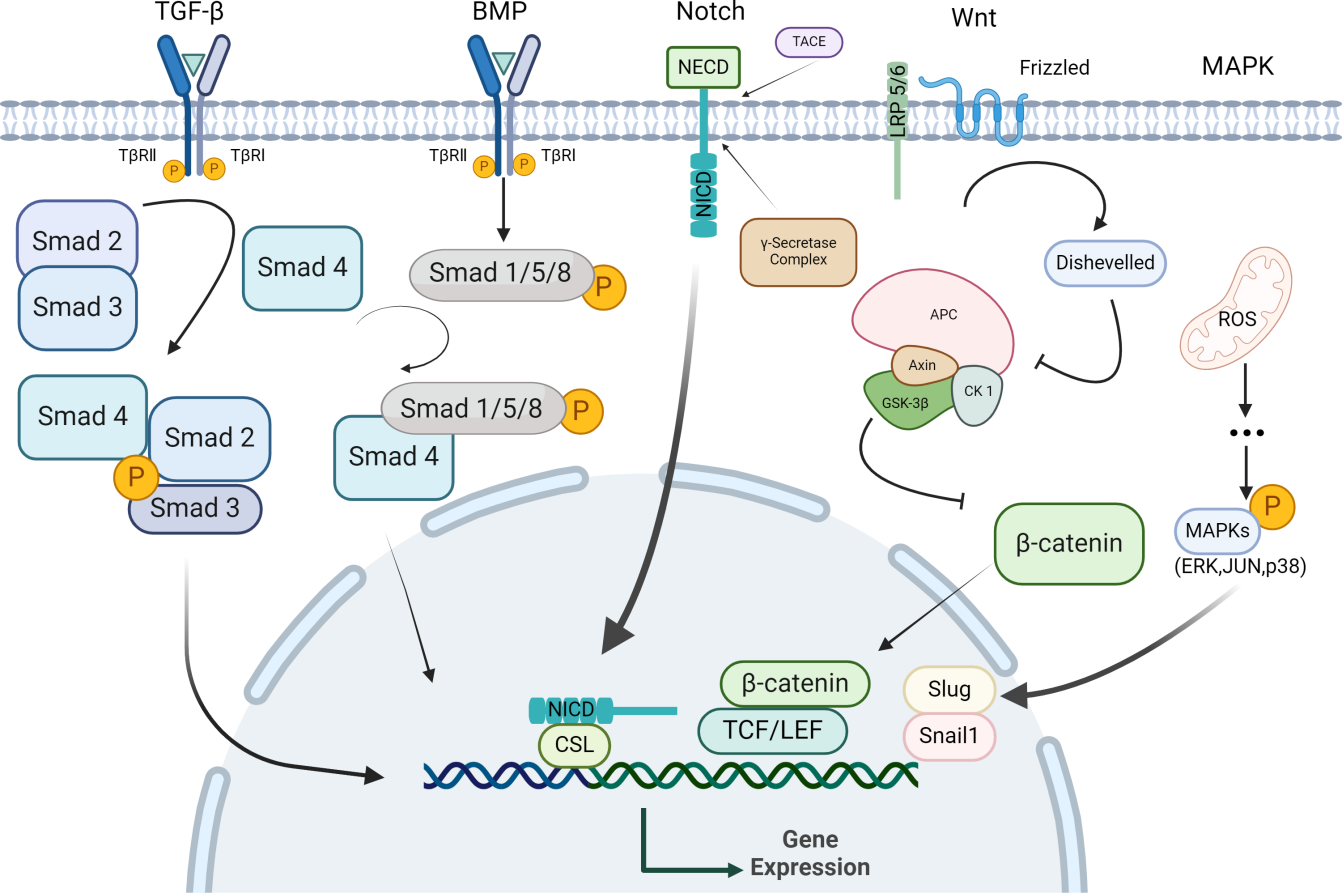
Signaling pathway regulating EMT in epithelial cells. Epithelial cells activate specific signaling pathways upon binding with their corresponding ligands. In response to these stimuli, epithelial cells undergo a series of cytoplasmic reactions that lead to the formation of activation complexes, these complexes then translocate to the nucleus, where they regulate target genes expression by repressing epithelial-specific genes and upregulating stromal-specific genes, thereby facilitating EMT.

The key steps in the EMT process encompass: 1) the disruption of tight junctions between epithelial cells, 2) the reshaping of cytoskeleton, and 3) epithelial-to-mesenchymal transition, characterized by downregulation of epithelial markers (E-Cadherin, claudins) and upregulation of mesenchymal markers (N-Cadherin, Vimentin, α-SMA, Snail, Zeb), which regulate cell migration potential (7). The EMT process can be regulated by various signaling pathways, including TGF-β, Wnt and Notch (7) (**Figure 1**). Some of the transcription factors also contribute to EMT, such as GATA4 and PRRX1 (26). Furthermore, the negative feedback loop involving transcription factors and microRNA and autophagy can also regulate EMT (27–29).

### 4.1 Signaling pathway regulation

#### 4.1.1 TGF-β-mediated signaling pathway

TGF-β is a multifunctional cytokine crucial for growth and development (30, 31). The TGF-β family comprises secreted polypeptides, such as TGF-β1 and bone morphogenetic proteins (BMPs) (32). TGF-β is essential for the maintaining cell homeostasis, development and numerous physiological functions in the lungs and other organs. It is also implicated in the pathogenesis of various respiratory diseases, including pulmonary fibrosis, chronic obstructive pulmonary disease, and asthma (33). Zhang et al. demonstrated that in asthma, airway epithelial cells undergo EMT and differentiation into myofibroblasts under TGF-β1 stimulation (34). This process contributes to the accumulation of myofibroblast in the subepithelial fibrosis stage of the airway. Doerner et al. found that TGF-β1 induced EMT in bronchial epithelial cells in a dose-dependent manner (3). Given that airway fibrosis correlates with increase of TGF-β1 expression, airway EMT may contribute to the airway remodeling process in asthma. Hackett et al. reported that EMT was prevalent in air-liquid interface cultures from asthmatic patients, with greater number of cells undergoing EMT compared to normal subjects (35). Furthermore, the study indicated that the primary airway epithelial cells from asthmatic patients undergo EMT via TGF-β1/Smad3 signaling. These findings suggest that TGF-β1 may facilitate airway epithelial fibrosis by modulating EMT and accelerating the airway remodeling in asthma.

#### 4.1.2 Bone forming protein pathway

Bone morphogenetic proteins (BMPs) are highly conserved members of the TGF-β cytokine superfamily (36). BMPs primarily rely on the classical Bmp/Smad signaling pathway to mediate biological effects (37). In the Bmp/Smad signaling pathway, ligand maturation proteins form homologous/heterodimers, which sequentially are activated and bind to SMAD4, forming SMAD protein heterocomplexes, these complexes then translocate to the nucleus and bind to target genes to regulate transcription (37, 38). Evidence suggests that the BMP signaling pathway is also implicated in the process of EMT in airway remodeling. A study by McCormack et al. demonstrated that exogenous bone morphogenetic proteins (BMP2, BMP4, BMP7) can induce EMT in normal airway epithelial cells of rats (39). This induction may enhance cell mobility by altering the expression of EMT-related proteins. However, BMP antagonists can inhibit cell migration and wound repair. It is suggested that BMP signaling pathway mediates the EMT process and may play a role in the development of airway remodeling. Thus, the BMP pathway may serve as a potential therapeutic target for modulating EMT in asthma.

#### 4.1.3 Wnt signaling pathway

The Wnt/β-catenin signaling pathway is crucial for cell growth, proliferation and maintenance of homeostasis. Excessive constriction or dilation of bronchi can activate β-catenin, leading to its involvement in tissue repair and the promotion of fibrosis in airway epithelium through mechanisms such as cell proliferation and production of differentiated extracellular matrix (40). In the classical Wnt signaling pathway, activation of Wnt/β-catenin signaling occurs when the Wnt ligand binds to the coiled protein (FZD) receptor and the low-density lipoprotein receptor-associated 5/6 (LRP5/6) co-receptor on the target cell, leading to the recruitment of the disheveled protein (Dvl). Activation of Dvl triggers a signaling cascade that destabilizes the multiprotein complex composed of glycogen synthase kinase 3β (GSK-3β), casein kinase 1 (CK-1), Axin, and adenomatous polyposis coli (APC). Consequently, cytoplasmic β-catenin accumulates, and the excess, active β-catenin translocates to the nucleus, where it functions as a transcriptional co-activator of T cell factor (TCF)/lymphatic enhancer (LEF) to activate Wnt target genes (40). Numerous studies have demonstrated that the Wnt/β-catenin signaling pathway is integral to the onset and progression of asthma. Research by Huo et al. demonstrated that blocking β-catenin not only alleviates ovalbumin OVA-induced airway inflammation in asthmatic rats by modulating pro-inflammatory and anti-inflammatory cytokines balance but also mitigates its pro-fibrotic effect associated with pathological airway remodeling by inhibiting EMT (41). Song et al. found that inhibition of Wnt/β-catenin signaling pathway significantly reduced airway remodeling and inhibited EMT in the airway epithelium of asthmatic rats (42). These studies suggest that the Wnt signaling pathway exacerbates airway inflammation through pro-inflammatory mechanisms, and promotes airway EMT, thereby intensifying airway remodeling in asthma.

#### 4.1.4 Notch signaling pathway

Notch is also a crucial regulator of EMT induction. Upon interaction with Notch ligands from neighboring cells, γ-secretase and TACE cleave Notch intracellular domain (NICD), facilitating its translocation into the nucleus where it forms activated protein complexes that promote downstream gene transcription (43, 44). Notch signaling directly regulates Snail1 expression (45, 46), and also exerts an indirect effect by inducing hypoxia-inducible factor-1α (HIF-1α). HIF-1α binds to the promoter of Lyamino oxidase (LOX), regulating its transcription and subsequent LOX-mediated Snail1expression (47). The expression of Snail1 in the epithelium is essential for regeneration following lung injury. Snail1 interacts with Notch, which is vital for Notch-mediated alterations in the expression of EMT marker proteins, such as E-cadherin and α-SMA (48). Volckaert et al. demonstrated that, following lung injury, activated Notch signaling in Clara cells, a sub-population of epithelial cells, induces Snail1 expression, leading to the initiation of transient EMT for repair (49). Consequently, Clara cells can adopt an epithelial stem cell phenotype characterized by Snail1 expression. This finding is significant for elucidating the mechanisms underlying the dysregulation of lung injury repair in asthma. Additionally, *in vitro* and *in vivo* studies by Namba et al. demonstrated that activation of Notch-1 receptor-mediated signal transduction induced EMT, whereas silencing Notch1 can reverse this process (50). In addition to its direct effects mediated by the intracellular domain, Notch also indirectly regulates EMT through various signaling pathways and microRNAs (51). Furthermore, the Notch signaling pathway is one of the most important drivers of TGF-β-induced EMT, which can act in a synergistic manner with TGF-β (51, 52). In conclusion, the Notch signaling pathway can regulate the occurrence of EMT in a directly or indirectly manner.

#### 4.1.5 ROS/MAPK signaling pathway

Mitogen-activated protein kinase (MAPK) signaling pathways are categorized according to their involvement in distinct protein kinase cascades, including extracellular signal-associated kinase (ERK1/2), Jun amino-terminal kinase (JNK1/2/3), and P38-MAPK. MAPK pathways are highly responsive to oxidative stress (OS), increased OS production can activate these pathways, which play a role in the onset and progression of various diseases (53, 54). In an *in vitro* study of EMT induced by TGF-β1, Ge et al. found that increased reactive oxygen species (ROS) levels in epithelial cells led to alterations in EMT marker proteins, such as α-SMA, N-cadherin and E-cadherin, Additionally, ROS/MAPK signaling pathway were found to inhibit TGF-β1-induced EMT (55). These findings suggest that the ROS/MAPK signaling pathway may be involved in EMT of the asthmatic airway.

### 4.2 MicroRNA regulation

MicroRNAs (miRNAs) have been extensively studied for their roles in regulating the pathological processes of asthma (56–58). Recent studies have demonstrated that miRNAs serve as effective regulators of EMT and mesenchymal-to-epithelial transition (MET), integral components of post-transcriptional regulation. These molecules can target multiple components that influence epithelial integrity and mesenchymal traits, thereby regulating the expression of key proteins involved in these processes. Additionally, miRNAs are implicated in the regulation of stem cell pluripotency and the modulation of tumor progression by influencing EMT and MET processes (59–61). *In vivo* and *in vitro* studies conducted by Liu et al. revealed that the expression levels of miR-106b-5p in asthmatic mice and TGF-β1-induced epithelial cells were negatively correlated with pulmonary fibrosis and EMT in asthma (62). The miR-106B-5p/E2F1/SIX1 signaling pathway may represent a potential therapeutic target for asthma. Furthermore, a study by Yang et al. found that miR-448-5p was significantly downregulated in lung tissue and TGF-β1-stimulated bronchial epithelial cells of asthmatic mice (63). Overexpression of miR-448-5p led to decreased expression of SIX1, subsequently inhibiting TGF-β1-mediated EMT and fibrosis. Thus, the miR-448-5p/SIX1 signaling may play a critical role in the process of EMT and subepithelial fibrosis associated with asthma. An *in vitro* study by Fan et al. showed that the miR-203a-3p/SIX1 signaling pathway axis can modulate EMT in TGF-β1-induced asthma models through Smad3 pathway (64). These studies collectively confirmed that miRNAs are involved in the regulation of EMT in asthma.

### 4.3 Autophagy regulation

Autophagy is the process by which cytoplasmic macromolecules or organelles are encapsulated into vesicles and subsequently fused with lysosomes (65). Autophagy plays a critical role in the pathogenesis and progression of numerous diseases through diverse regulatory mechanisms (66). The regulatory pathways associated with autophagy significantly influence EMT, which is itself critically regulated by autophagic processes (67). Research conducted by Liu et al. demonstrated that asthmatic mice exhibited an increased number of autophagosomes compared to healthy controls, indicating that autophagy is implicated in the induction of EMT and airway remodeling in asthma (28). Cho et al. proposed that epithelial autophagy, initiated by oxidative stress, regulates airway epithelial fibrosis through induction of EMT processes (68). Furthermore, accumulating evidence indicates that the functional interaction between cytoskeleton and mitochondria serves as a pivotal regulatory mechanism in both autophagy and EMT processes (67).

## 5 Conclusion and future prospects

In summary, the repair of airway injury resulting from chronic inflammation in asthma involves interstitial transformation of airway epithelial cells mediated by specific signaling pathways. This transformation induces alterations in extracellular matrix deposition and airway fibrosis, ultimately contributing to structural remodeling of the airway walls. Recent studies indicate that EMT significantly contributes to both lung development and the pathophysiology of asthma. Currently studies have shown that certain pharmacological agents can inhibit tumor growth and metastasis by modulating the expression of EMT-related protein markers (69, 70). Additionally, studies reveal that specific drugs can target gene expression involved in EMT in asthma, enhancing the expression of epithelial markers and subsequently improving the barrier function at inflammatory sites. This suggests promising pharmacological avenues for asthma treatment (13, 71). Despite progress in understanding the epithelial-mesenchymal transition (EMT) in asthma and establishing a theoretical framework, several unresolved questions remain:1) Molecular mechanisms of EMT: While specific signaling pathways, including the TGF-β1, Wnt/β-catenin and sonic hedgehog pathways, are known to be involved in EMT in asthma, the interactions between these pathways, their regulatory networks and the precise mechanisms controlling EMT remain unclear. 2) Triggers of EMT: Chronic inflammation is thought to be a major trigger of EMT in asthma, but the specific inflammatory mediators, cytokines, and their interactions with airway epithelial cells to initiate EMT require further investigation. 3) EMT and asthma severity: Although EMT is strongly associated with airway remodeling and asthma severity, whether its manifestation, rate of progression and response to treatment differ in asthmatic patients remains to be investigated. 4) Reversibility of EMT: Some studies suggest that certain drugs can inhibit or reverse EMT, but the mechanisms, long-term effects and clinical application of these treatments in asthma require further research and validation. 5) Genetic and epigenetic regulation of EMT in asthma: While environmental factors (e.g. allergens, pollutants) influence EMT, genetic predisposition and epigenetic modifications may also play an important role. Understanding how genetic mutations, epigenetic changes or alterations in gene expression contribute to the initiation and maintenance of EMT in asthma may provide new insights for personalized therapies. 6) EMT and asthma complications: Asthma patients often have comorbidities such as chronic obstructive pulmonary disease (COPD) and bronchiectasis. The potential relationship between these complications and EMT, as well as the role of EMT in their development, warrants further investigation. Addressing these unresolved issues is essential for advancing our understanding of asthma pathogenesis and for the development of targeted therapies that can modulate EMT and its associated processes in the context of asthma.
